# Supplementary material for: Evaluation of the Heat Shock Protein 90 Inhibitor Ganetespib as a Sensitizer to Hyperthermia-Based Cancer Treatments
Source: Cancers (Basel). 2022 Oct 26;14(21):5250. doi: 10.3390/cancers14215250 (PMC9654690; doi:10.3390/cancers14215250)
Supplement: Supplementary file 1 [file cancers-14-05250-s001.zip › cancers-1972052-supplementary.pdf]

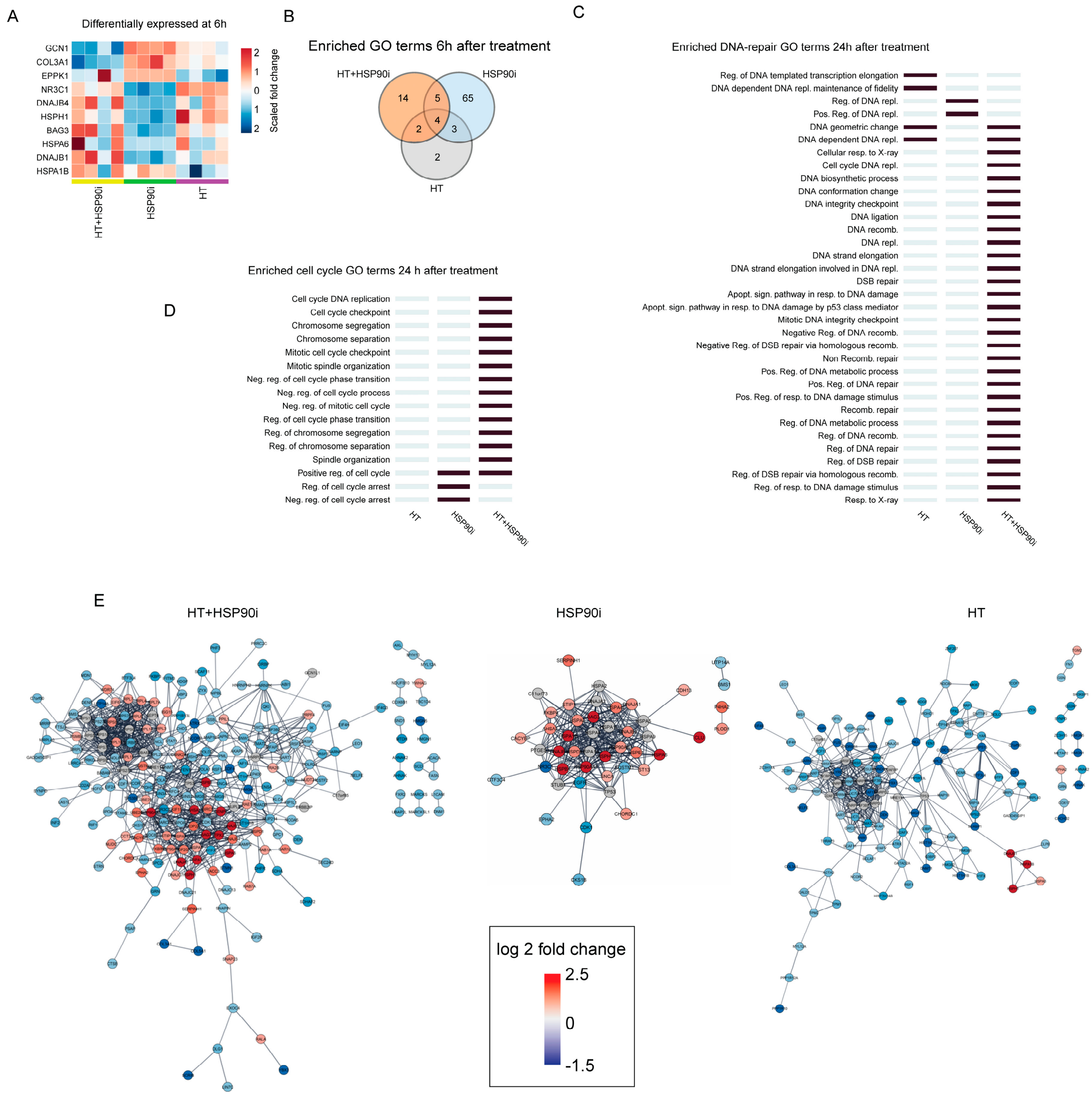

Figure S1. Proteome alterations caused by ganetespib and hyperthermia. (A) Heatmap of proteins found to be up- or downregulated in at least one condition 6 hours after hyperthermia (HT). (B) Venn diagram of gene ontology (GO) terms found to be enriched per condition 6 hours after HT. (C-D) Heatmaps displaying enriched DNA-repair and cell cycle GO terms 24 hours after HT (marked in dark blue). (E) Protein-protein interaction networks of up- and downregulated proteins 24 hours after HT.

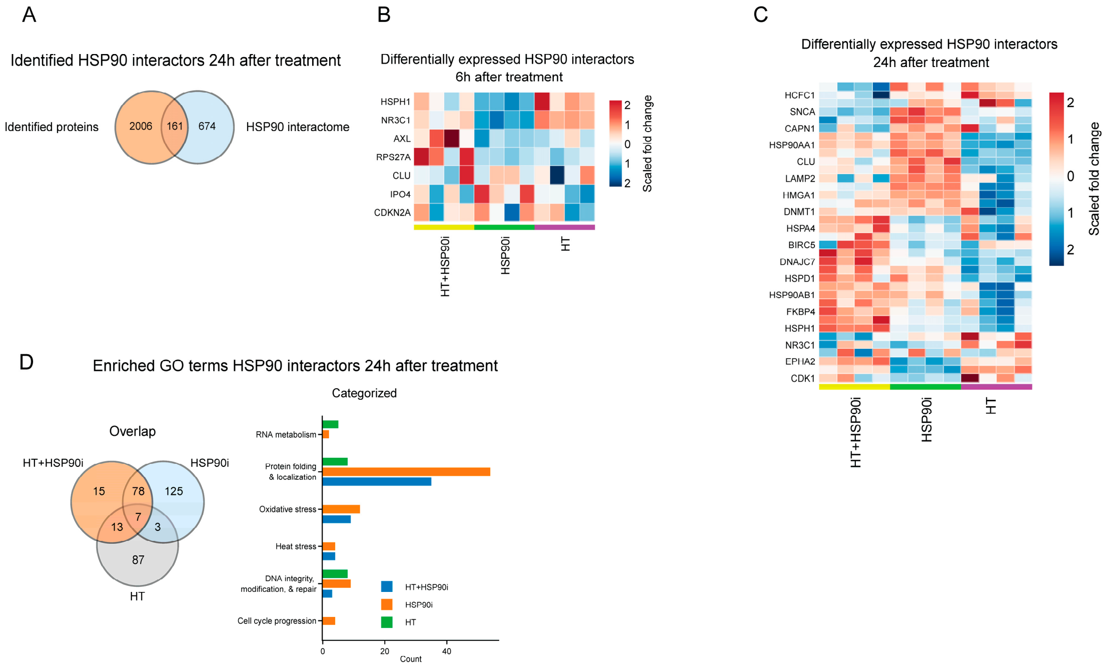

Figure S2. Proteome alterations caused by ganetespib and hyperthermia. (A) Venn diagram showing identified HSP90 interactors in the dataset. (B-C) Heatmaps of interactors found to be up- or downregulated in at least one condition at 6 and 24 hours after hyperthermia (HT). (D) Venn diagram and hand-curated categories of gene ontology (GO) terms that were found to be enriched.

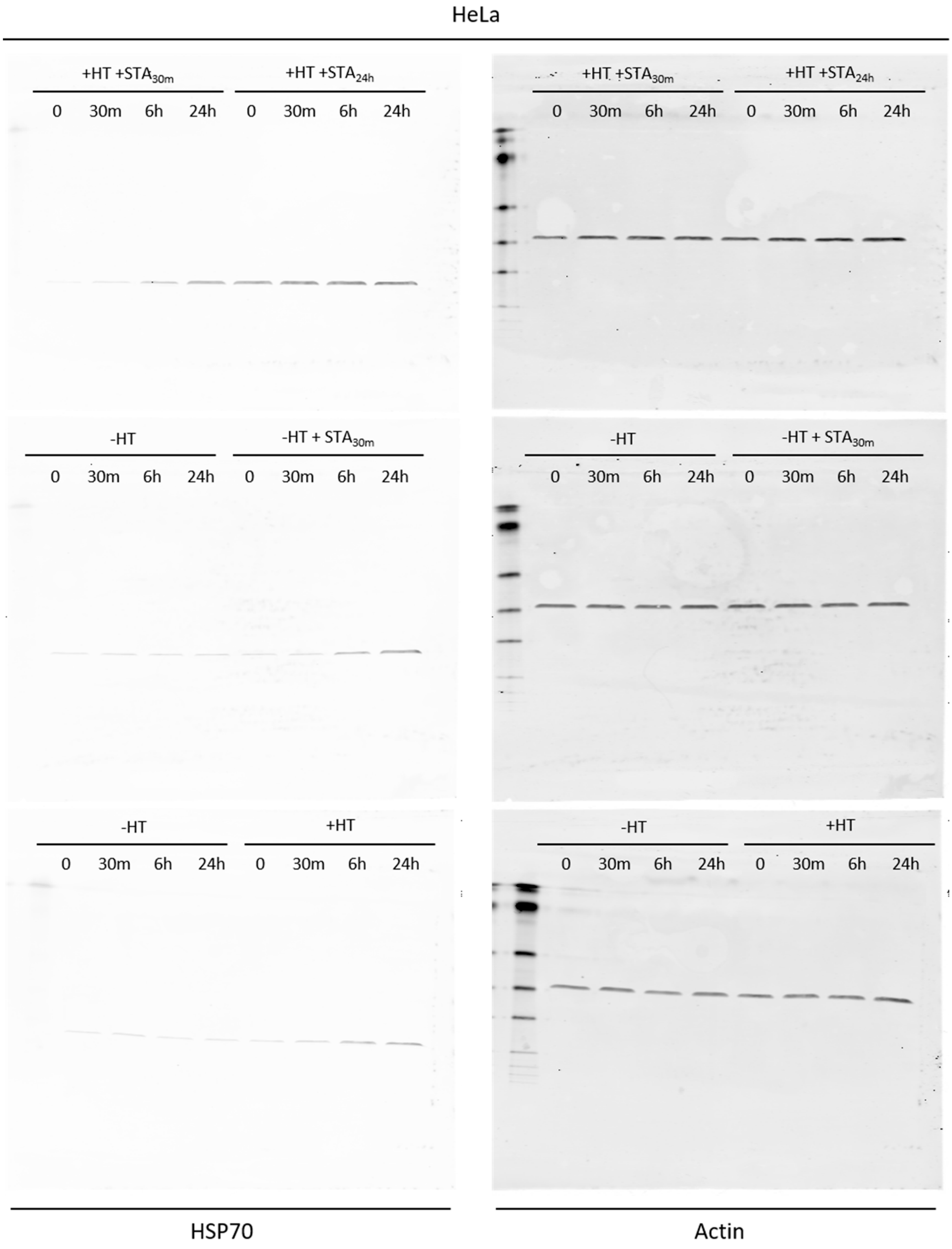
HeLa
+HT +STA30m
+HT +STA24h
0
30m
6h
24h
-HT
-HT + STA30m
+HT
HSP70
Actin
(**a**)

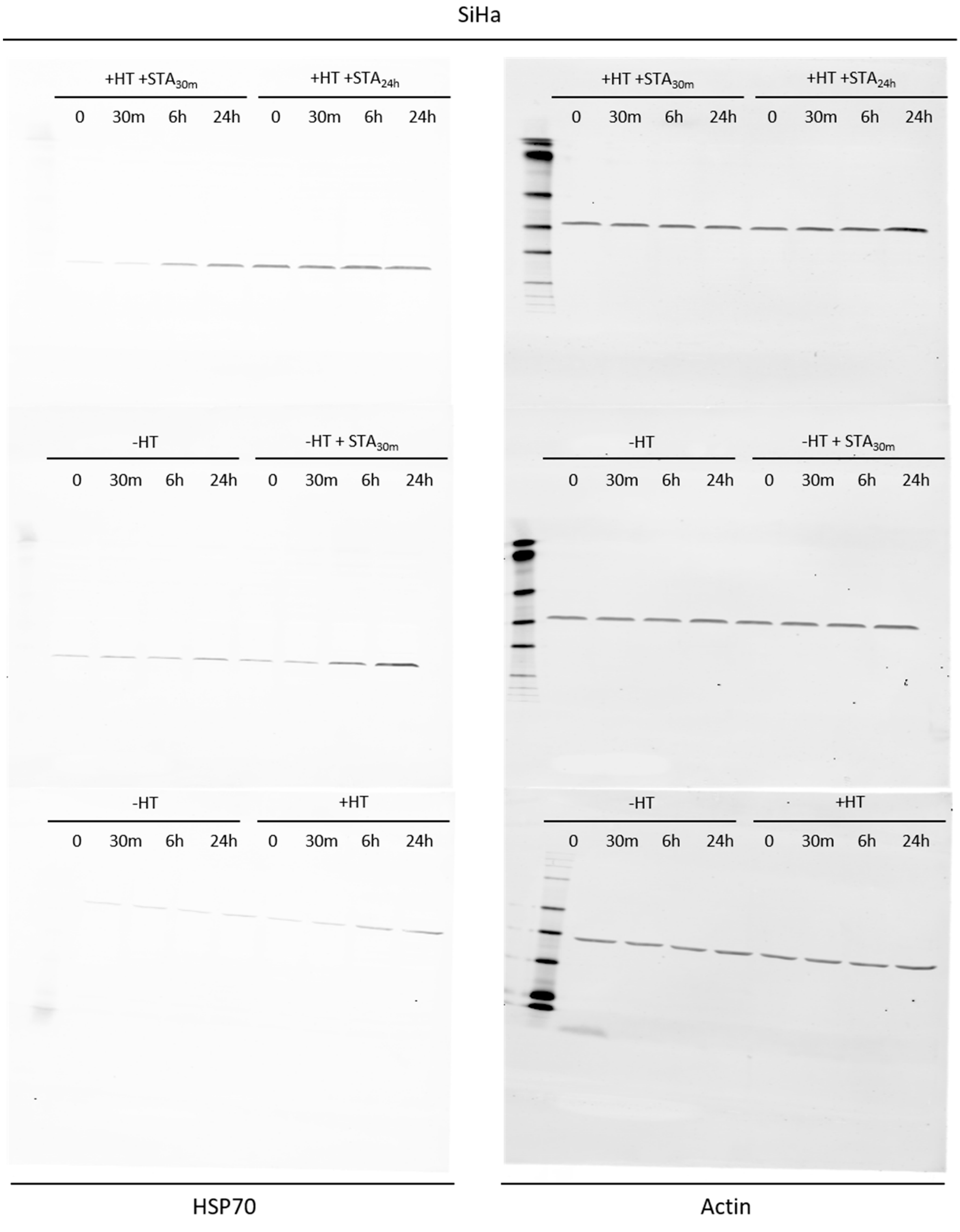

(**b**)

Figure S3. (**a**) Raw data of HeLa HSP70 actin; (**b**) Raw data of SiHa HSP70 actin.

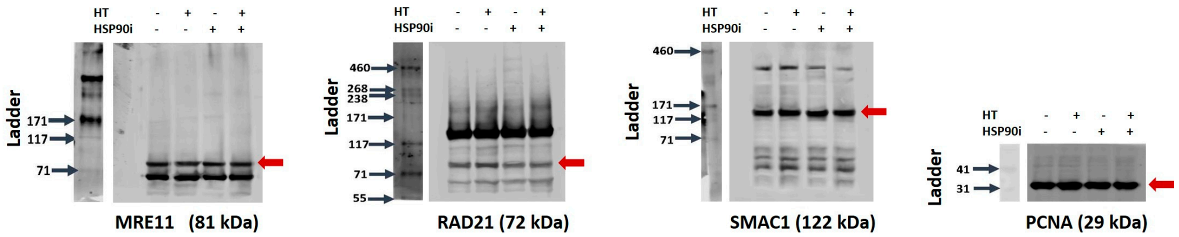

Figure S4. Raw image of annotated Figure 4H.
